# Structure–activity characteristics of phenylalanine analogs selectively transported by L-type amino acid transporter 1 (LAT1)

**DOI:** 10.1038/s41598-024-55252-w

**Published:** 2024-02-26

**Authors:** Sihui Chen, Chunhuan Jin, Ryuichi Ohgaki, Minhui Xu, Hiroki Okanishi, Yoshikatsu Kanai

**Affiliations:** 1 Department of Bio-System Pharmacology, Graduate School of Medicine, Osaka University, 2-2, Yamadaoka, Suita, Osaka 565-0871 Japan; 2 Integrated Frontier Research for Medical Science Division, Institute for Open and Transdisciplinary Research Initiatives (OTRI), Osaka University, Suita, Osaka 565-0871 Japan; 3 Premium Research Institute for Human Metaverse Medicine (WPI-PRIMe), Osaka University, Suita, Osaka 565-0871 Japan

**Keywords:** Drug delivery, Cancer

## Abstract

L-type amino acid transporter 1 (LAT1) is a transmembrane protein responsible for transporting large neutral amino acids. While numerous LAT1-targeted compound delivery for the brain and tumors have been investigated, their LAT1 selectivity often remains ambiguous despite high LAT1 affinity. This study assessed the LAT1 selectivity of phenylalanine (Phe) analogs, focusing on their structure–activity characteristics. We discovered that 2-iodo-l-phenylalanine (2-I-Phe), with an iodine substituent at position 2 in the benzene ring, markedly improves LAT1 affinity and selectivity compared to parent amino acid Phe, albeit at the cost of reduced transport velocity. l-Phenylglycine (Phg), one carbon shorter than Phe, was found to be a substrate for LAT1 with a lower affinity, exhibiting a low level of selectivity for LAT1 equivalent to Phe. Notably, (*R*)-2-amino-1,2,3,4-tetrahydro-2-naphthoic acid (bicyclic-Phe), with an α-methylene moiety akin to the α-methyl group in α-methyl-l-phenylalanine (α-methyl-Phe), a known LAT1-selective compound, showed similar LAT1 transport maximal velocity to α-methyl-Phe, but with higher LAT1 affinity and selectivity. In vivo studies revealed tumor-specific accumulation of bicyclic-Phe, underscoring the importance of LAT1-selectivity in targeted delivery. These findings emphasize the potential of bicyclic-Phe as a promising LAT1-selective component, providing a basis for the development of LAT1-targeting compounds based on its structural framework.

## Introduction

Targeted drug delivery aims to deliver drugs to specific sites within the body at effective concentrations, maximizing therapeutic effects while minimizing adverse reactions due to drug accumulation at non-target sites^1,2^. The recent focus on transporter-targeted drug delivery leverages transporters that are predominantly expressed in target tissues^3^. Drugs are designed to be substrates of these transporters, enabling selective delivery to specific tissues^4^. Among these transporters, L-type amino acid transporter 1 (LAT1, SLC7A5) is particularly promising for targeted drug delivery, given its tissue-specific expression profile^5^. As an isoform of the amino acid transport system L, LAT1 transports large neutral amino acids with branched or aromatic side chains, such as leucine, valine, isoleucine, methionine, phenylalanine, tyrosine, tryptophan, and histidine^6–8^. Another isoform, LAT2 (SLC7A8), shares similar substrate selectivity but also transports smaller neutral amino acids, including glycine, alanine, serine, threonine, cysteine, asparagine, and glutamine^9^. While LAT2 is ubiquitously distributed in normal tissues, particularly in renal proximal tubules and small intestinal epithelium, LAT1 is highly expressed in various cancers and is also present in the blood–brain barrier (BBB) and placental barrier^5,9–18^. In the placenta, LAT1 is predominantly expressed in the syncytiotrophoblast layer, which is critical not only for transporting amino acids across the placental barrier but also for the development of the syncytiotrophoblast itself. Mice with a homozygous knockout of the LAT1 gene exhibit embryonic lethality due to abnormalities in placental formation^18^. Furthermore, a study has shown that homozygous mutations in the LAT1 gene, affecting its function in the BBB maintaining branched-chain amino acids pivotal to brain development, may present with the symptoms associated with an autism spectrum disorder^19^. Taking advantage of its characteristic expression, LAT1 has been exploited for drug delivery targeting both cancers and the brain. An ideal drug for LAT1-targeted delivery should possess high affinity and selectivity for LAT1, along with efficient transport velocity.

LAT1 is expressed in brain capillary endothelial cells forming BBB^15,16^. The tight junctions between these cells restrict most drug permeation, posing a challenge in achieving effective drug concentrations in the brain, thereby limiting therapeutic efficacy^20^. Drugs such as gabapentin^21^, an anticonvulsant, and l-DOPA^17^, an antiparkinsonian drug, are designed to mimic LAT1's endogenous substrates structurally. This strategy utilizes LAT1 for enhanced delivery through the BBB to improve brain uptake. Gabapentin and l-DOPA have been shown to act as LAT1 substrates, though they possess relatively lower affinity compared to endogenous substrates like Phe^21–24^. In vivo studies revealed that a high protein diet or the infusion of large amounts of neutral amino acids before administering l-DOPA can reduce its transport to the brains of monkeys, implying that endogenous amino acids compete with l-DOPA for LAT1-mediated transport and high concentrations of these amino acids can reduce brain uptake of l-DOPA^25^. Hence, enhancing a drug's LAT1 affinity could improve its brain uptake. Furthermore, l-DOPA's low selectivity for LAT1 suggests that increasing its specificity for LAT1 may also boost its effectiveness in the brain^26^.

Due to its elevated expression in cancer tissues, LAT1 has emerged as a crucial target for compound delivery in cancer detection and therapy^5^. Positron emission tomography (PET) for imaging malignant tumors has been widely used for cancer detection^27,28^. 3-Fluoro-l-α-methyltyrosine (FAMT) has been developed as a LAT1-specific PET probe. Clinical PET studies using ^18^F-FAMT probe have shown high tumor accumulation with minimal uptake in normal or inflammatory tissues, thereby enhancing the accuracy of cancer detection^29–32^. Additionally, a series of fluoroethyl Phe analogs have recently been developed as LAT1-targeted PET tracers. Among them, some include substitutions with fluoroethyl groups at the *ortho* (position 2), *meta* (position 3), and *para* (position 4) positions in the benzene ring of Phe, each imparting distinct LAT1 affinities^33^. Notably, the *ortho* substitution exhibits the highest LAT1 affinity, but this comes at the expense of a reduced transport velocity^33^. It is hypothesized that the increased bulkiness of aromatic side chains is responsible for this reduction in LAT1 transport velocity^22,23^. Consequently, modifying the *ortho* fluoroethyl group to a smaller substituent could preserve high LAT1 affinity while minimizing its impact on transport velocity. Halogen groups at the *meta* position in the benzene ring of Phe derivatives enhance LAT1 affinity in proportion to their size while having minimal effect on the transport velocity^34^. However, the effects of different halogen groups at the *ortho* and *para* positions on LAT1 affinity, selectivity, and transport velocity warrant further investigation.

LAT1's role in cancer therapy drug delivery is also highlighted by melphalan (Phe-mustard), an alkylating agent selectively transported by LAT1 due to its bulky side chain^22,35,36^, although its transport velocity is relatively low^22–24^. To improve LAT1 transport efficiency, various amino acid-mustards have been synthesized, including Phg-mustard, which is one carbon shorter than melphalan and more effective as a substrate^37^. A further derivative of melphalan is dl-2-amino-7-bis[(2-chloroethyl)amino]-1,2,3,4-tetrahydro-2-naphthoic acid, known as bicyclic Phe-mustard, in which nitrogen mustard is attached to the *meta* position of the aromatic ring in bicyclic-Phe^38^. This compound has been found to enhance its interaction with system L significantly^38^. However, the LAT1 selectivity of these amino acid-mustards remains a concern, as the distribution of nitrogen mustard in normal tissues can lead to serious side effects, such as myelosuppression^39^.

In this study, we first explored the structural preferences for LAT1 affinity among halogenated phenylalanines. We then conducted quantitative analyses to assess LAT1 affinity (*K*_i_), selectivity (*K*_i_ ratio of LAT2 to LAT1), and transport velocity (*V*_max_ of efflux) of these Phe analogs, aiming to delineate the structural features critical for LAT1-targeted compound delivery. Subsequent in vivo experiments further validated the role of LAT1 selectivity in the biodistribution of the compounds. The insights gained from this study would establish a foundation for the rational design and synthesis of LAT1-targeted compounds, potentially advancing drug delivery strategies that exploit LAT1 in the BBB and cancer cells.

## Results

### Interaction of halogenated phenylalanines with LAT1 and LAT2

**Figure 1:**
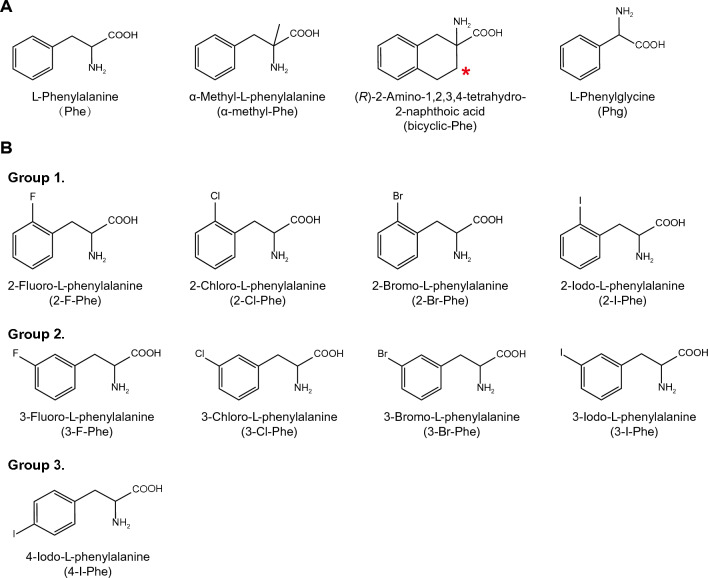
Chemical structures of Phe and its analogs used in this study. (**A**) The parent amino acid Phe, α-methyl-Phe, bicyclic-Phe (with its methylene group marked by an asterisk), and Phg. (**B**) Halogenated phenylalanines classified into three groups based on the position of the halogen in the benzene ring: Group 1 with halogens at position 2 *(ortho*-position), Group 2 with halogens at position 3 (*meta*-position), and Group 3 with halogens at position 4 (*para*-position).

**Figure 2:**
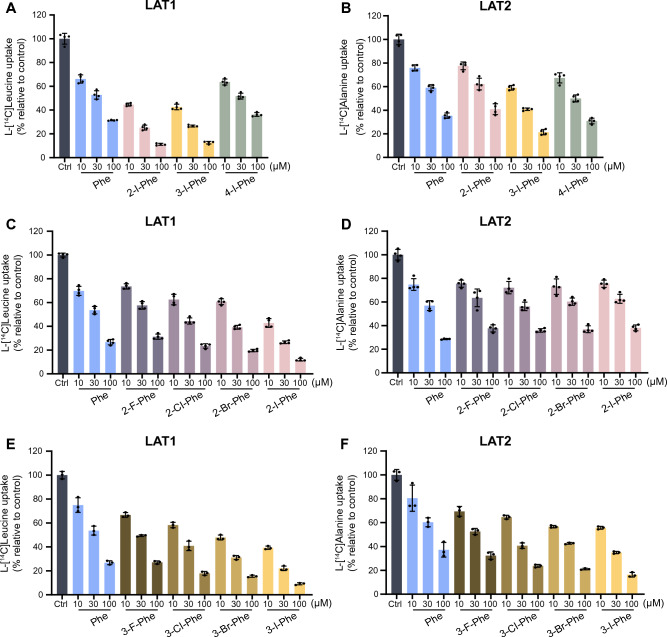
LAT1 and LAT2 selectivity of halogenated phenylalanines. (**A**) Inhibition of LAT1-mediated l-[^14^C]leucine uptake by iodinated Phe isomers (positions 2, 3, and 4) in HEK293-hLAT1 cells. The uptake of l-[^14^C]leucine (1 μM) was measured for 1 min in the absence (control, Ctrl) or presence of Phe and iodinated Phe isomers at 10, 30, and 100 μM. (**B**) Inhibition of LAT2-mediated l-[^14^C]alanine uptake by iodinated Phe isomers in HEK293-hLAT2 cells. Similar to (**A**), uptake was measured in the absence (control, Ctrl) or presence of the Phe and iodinated Phe isomers at 10, 30, and 100 μM. (**C** and **D**) Inhibitory impact of *ortho*-position (position 2) halogenated phenylalanines on l-[^14^C]leucine uptake in HEK293-hLAT1 cells (**C**) and l-[^14^C]alanine uptake in HEK293-hLAT2 cells (**D**). (**E** and **F**) Examination of the inhibitory effects of *meta*-position (position 3) halogenated phenylalanines on l-[^14^C]leucine uptake in HEK293-hLAT1 cells (**E**) and l-[^14^C]alanine uptake in HEK293-hLAT2 cells (**F**). The uptake values are presented as the percentage of l-[^14^C]leucine or l-[^14^C]alanine uptake relative to control (Ctrl). Data are expressed as mean ± S.D., n = 3–4.

In this study, we used the HEK293 cells stably expressing human LAT1 or human LAT2, which form a heterodimeric complex with endogenous 4F2hc (CD98hc), required for plasma membrane localization^26^. We first explored the impact of halogen group positioning in the benzene ring of Phe (Fig. 1) on its interaction with LAT1 and LAT2. This approach was prompted by earlier studies suggesting that the location of substituents in aromatic rings of LAT1 ligands influences their affinity to LAT1^33,34^. We conducted inhibition experiments to measure the uptake of l-[^14^C]leucine by LAT1 and l-[^14^C]alanine by LAT2 in the presence or absence of Phe derivatives with an iodo group at position 2, 3, or 4 in the benzene ring. As shown in Fig. 2A, LAT1-mediated uptake of l-[^14^C]leucine was markedly inhibited by 2-I-Phe and 3-I-Phe compared with the parent amino acid Phe, a well-established substrate of both LAT1 and LAT2. The inhibitory effect of 4-I-Phe on l-[^14^C]leucine uptake was comparable to that of Phe. In contrast, as shown in Fig. 2B, LAT2-mediated uptake of l-[^14^C]alanine was inhibited by Phe and its iodinated analogs, following a trend of 2-I-Phe = Phe < 4-I-Phe < 3-I-Phe. These results indicate that an iodo group at position 3 in the benzene ring increases the affinity to both LAT1 and LAT2, surpassing that of the parent compound Phe. On the other hand, an iodo group at position 2 increases the affinity for LAT1 but does not alter affinity for LAT2. These observations suggest that the introduction of a halogen group at position 2 in the benzene ring of Phe would impart the selective affinity towards LAT1.

The observations that an iodo group at either position 2 or 3 in the benzene ring enhances affinity for LAT1 have led to further investigation into the effects of other halogens at these positions, focusing on their interactions with both LAT1 and LAT2. When a halogen group is located at position 2 in the benzene ring, we observed an increase in LAT1 inhibition correlating with the size of the halogen: 2-F-Phe = Phe < 2-Cl-Phe < 2-Br-Phe < 2-I-Phe (Fig. 2C). However, the size of the halogen group did not significantly influence the inhibition of LAT2 (Fig. 2D). When the halogen group is at position 3, both LAT1 and LAT2 inhibition increased with the halogen size: Phe < 3-F-Phe < 3-Cl-Phe < 3-Br-Phe < 3-I-Phe (Fig. 2E,F). These findings suggest that the halogen size affects interactions with LAT1 and LAT2 differently; bulkier halogens at position 3 interact with both LAT1 and LAT2 with greater affinity, whereas larger halogens at position 2 enhance affinity to LAT1 without altering LAT2 interactions. Consequently, 2-I-Phe was chosen for further comparative studies with other Phe-related compounds due to its high LAT1 affinity and selectivity.

### LAT1 selectivity of Phe analogs

**Figure 3:**
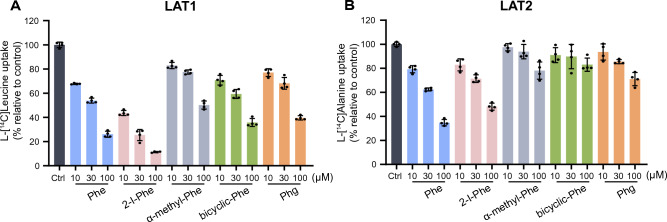
LAT1 and LAT2 selectivity of Phe and its analogs. Inhibition of LAT1-mediated l-[^14^C]leucine (1 μM) uptake (**A**) or LAT2-mediated l-[^14^C]alanine (1 μM) uptake (**B**) was measured in the absence (control, Ctrl) or presence of Phe and its analogs 2-I-Phe, α-methyl-Phe, bicyclic-Phe, and Phg at 10, 30, and 100 μM. The uptake values are expressed as the percentage of l-[^14^C]leucine or l-[^14^C]alanine uptake relative to control (Ctrl). Data are presented as mean ± S.D., n = 4.

**Figure 4:**
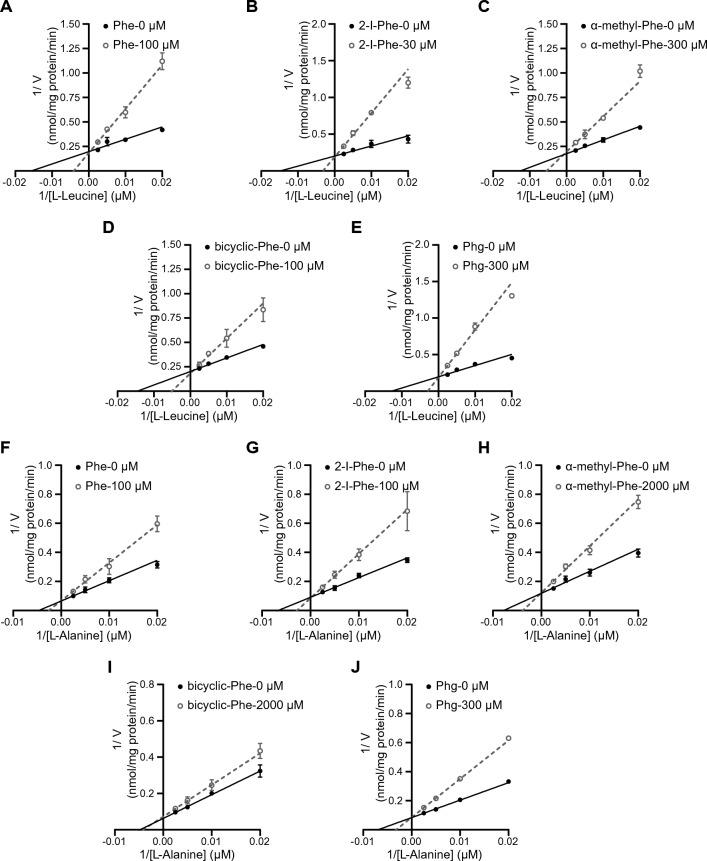
Inhibition kinetics of Phe analogs on LAT1 and LAT2. This figure presents Lineweaver–Burk plots to analyze the inhibition kinetics of Phe and its analogs on l-[^14^C]leucine uptake in HEK293-hLAT1 cells (**A**-**E**) and l-[^14^C]alanine uptake in HEK293-hLAT2 cells (**F**-**J**). The uptake of l-[^14^C]leucine or l-[^14^C]alanine (50, 100, 200, and 400 μM) was measured in the absence (closed circles) or presence of Phe and its analogs (open circles). Data are expressed as mean ± S.D., n = 3–4.

In the inhibition experiments assessing the interaction of Phe and its analogs with LAT1 and LAT2, Phe, 2-I-Phe, α-methyl-Phe, bicyclic-Phe, and Phg were all found to inhibit LAT1-mediated l-[^14^C]leucine uptake (Fig. 3A). In contrast, α-methyl-Phe, bicyclic-Phe, and Phg showed minimal inhibition of LAT2-mediated l-[^14^C]alanine uptake at the concentrations tested (Fig. 3B). We further undertook a quantitative analysis to assess the inhibitory properties and LAT1 selectivity of the Phe analogs. The uptake of l-[^14^C]leucine by LAT1 and l-[^14^C]alanine by LAT2 was quantified in the presence or absence of Phe and its analogs, with the data double-reciprocally fitted to Lineweaver–Burk plots. As shown in Fig. 4, all tested compounds displayed competitive inhibition in Lineweaver–Burk plots. *K*_i_ values, independent of compound concentrations (Supplementary Fig. S1), are presented in Table 1. Affinities for LAT1 and LAT2 were assessed based on the *K*_i_ values. LAT1 selectivity was quantified as the *K*_i_ ratio, calculated as LAT2 *K*_i_ over LAT1 *K*_i_. As indicated in Table 1, α-methyl-Phe, known for its LAT1 selectivity, showed a pronounced preference for LAT1 over LAT2, corroborating the findings from previous studies^26^. The *K*_i_ ratio for 2-I-Phe, similar to that of α-methyl-Phe, underscores its high LAT1 selectivity. Notably, bicyclic-Phe exhibited a higher *K*_i_ ratio, suggesting greater LAT1 selectivity than the other compounds. In contrast, the *K*_i_ ratio of Phg paralleled that of Phe, indicating that Phg does not exhibit enhanced LAT1 selectivity compared to Phe.

**Table 1:** *K*_i_ values of Phe analogs for LAT1 and LAT2. *K*_i_ values were determined as described in Materials and methods.

Compounds	*K*_i_ (μM)		*K*_i_ ratio (LAT2/LAT1)
	LAT1	LAT2	
Phe	43.47 ± 6.28	109.79 ± 20.0	2.53
2-I-Phe	9.56 ± 1.67	87.61 ± 11.87	9.16
α-methyl-Phe	156.57 ± 19.51	1681.67 ± 271.97	10.74
bicyclic-Phe	78.18 ± 13.94	3750.87 ± 740.05	47.98
Phg	96.80 ± 13.17	241.93 ± 20.80	2.50

Data are represented as mean ± S.E.M., n = 3–4.

### Transport of 2-I-Phe, α-methyl-Phe, and bicyclic-Phe by LAT1

**Figure 5:**
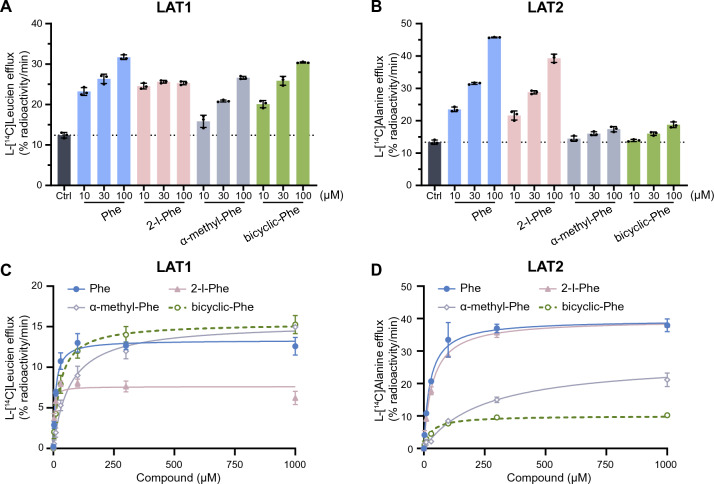
LAT1-mediated efflux of l-[^14^C]leucine and LAT2-mediated efflux of l-[^14^C]alanine induced by Phe analogs. (**A** and **B**) LAT1-mediated efflux of pre-loaded l-[^14^C]leucine from HEK293-hLAT1 cells (**A**) and LAT2-mediated efflux of pre-loaded l-[^14^C]alanine from HEK293-hLAT2 cells (**B**) induced by Phe and its analogs were measured. The efflux assessment was conducted for 1 min in the absence (control, Ctrl) or presence of Phe, 2-I-Phe, α-methyl-Phe, and bicyclic-Phe at concentrations of 10, 30, and 100 μM. The efflux values are presented as the percentage of pre-loaded l-[^14^C]leucine or l-[^14^C]alanine. (**C** and **D**) The concentration dependence of l-[^14^C]leucine efflux from HEK293-hLAT1 cells (**C**) and l-[^14^C]alanine efflux from HEK293-hLAT2 cells (**D**) on the extracellularly applied Phe and its analogs is shown. The efflux was measured for 1 min in the absence (control, Ctrl) or presence of varying concentrations of Phe (closed circles), 2-I-Phe (closed triangles), α-methyl-Phe (open rhombus), and bicyclic-Phe (open circles) ranging from 3 to 1000 μM. Efflux values in the absence of test compound (Ctrl) were subtracted from those in the presence of test compounds and fitted to Michaelis–Menten curves. Data are presented as mean ± S.D., n = 3.

To examine whether 2-I-Phe, α-methyl-Phe, and bicyclic-Phe, identified as selective for LAT1 in inhibition experiments (Table 1), are also selectively transported by LAT1 as substrates, we conducted efflux experiments utilizing the exchange properties of LAT1 and LAT2^40^. We evaluated the efflux of pre-loaded l-[^14^C]leucine from LAT1 and pre-loaded l-[^14^C]alanine from LAT2 induced by extracellularly applied compounds. As shown in Fig. 5A, extracellular application of Phe and its analogs induced a notable efflux of l-[^14^C]leucine from LAT1, suggesting that 2-I-Phe, α-methyl-Phe, and bicyclic-Phe are transported by LAT1 as substrates, akin to Phe. In contrast, α-methyl-Phe and bicyclic-Phe elicited less l-[^14^C]alanine efflux from LAT2 compared to Phe, a known substrate of LAT2, indicating that α-methyl-Phe and bicyclic-Phe are not efficiently transported by LAT2. The efflux induced by 2-I-Phe was comparable to that of Phe in LAT2 (Fig. 5B).

We subsequently conducted a quantitative analysis of the transport properties of these Phe analogs, as assessed by efflux experiments for LAT1 and LAT2. The efflux of pre-loaded l-[^14^C]leucine from LAT1 (Fig. 5C) and pre-loaded l-[^14^C]alanine from LAT2 (Fig. 5D) induced by extracellularly applied compounds were measured in a concentration-dependent manner and fitted to Michaelis–Menten curves. The kinetic parameters are provided in Table 2. The *K*_m_ values from the efflux experiments for the Phe analogs showed a similar trend to the *K*_i_ values from the inhibition experiments, except that the *K*_m_ value for the efflux of bicyclic-Phe in LAT2 was unexpectedly low. The observed discrepancy may be due to the presence of a small amount of endogenous LAT1 in HEK293-LAT2 cells as discussed later. In LAT1, the *V*_max_ values for α-methyl-Phe and bicyclic-Phe were equivalent to that of Phe, whereas the *V*_max_ for 2-I-Phe was about half that of Phe (Fig. 5C and Table 2). In LAT2, the *V*_max_ for 2-I-Phe was similar to that of Phe, but α-methyl-Phe and bicyclic-Phe had lower *V*_max_ values, with bicyclic-Phe showing a *V*_max_ only a quarter of that of Phe (Fig. 5D and Table 2).

**Table 2:** Kinetic parameters of Phe analogs for LAT1 and LAT2. *K*_m_ and* V*_max_ values were determined as described in Materials and methods.

	Compounds	*K*_m_ of efflux (μM)	*V*_max_ of efflux (% radioactivity/min)	Ratio to *V*_max_ of Phe
LAT1	Phe	8.92 ± 1.08	13.32 ± 0.31	(1.00)
	2-I-Phe	2.79 ± 0.76	7.62 ± 0.30	0.57
	α-methyl-Phe	67.95 ± 7.39	15.45 ± 0.45	1.16
	bicyclic-Phe	27.28 ± 3.08	15.43 ± 0.40	1.16
LAT2	Phe	25.21 ± 2.43	39.60 ± 0.74	(1.00)
	2-I-Phe	35.34 ± 1.95	39.65 ± 0.44	1.00
	α-methyl-Phe	246.49 ± 23.00	27.28 ± 0.70	0.69
	bicyclic-Phe	29.96 ± 4.00	10.05 ± 0.27	0.25

Data are represented as mean ± S.E.M., n = 3.

### Differential accumulation of bicyclic-Phe and 2-fluoro-l-tyrosine (2-F-Tyr) in tumor tissues vs. small intestine in mice

**Figure 6:**
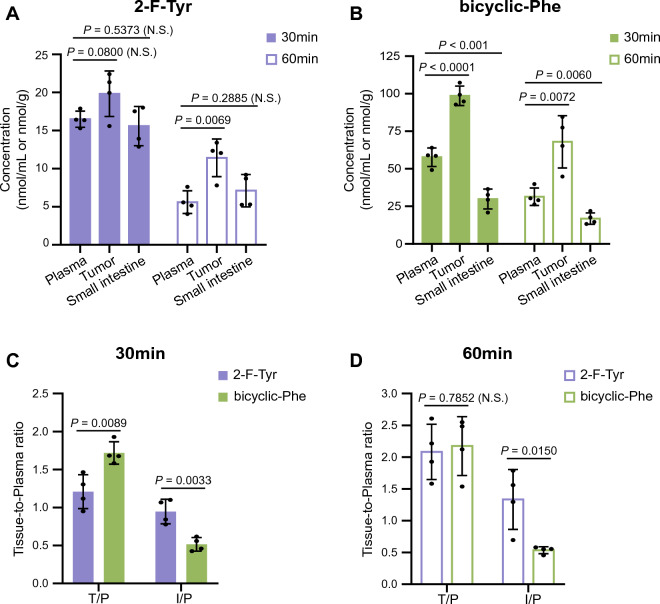
Tissue accumulation of 2-F-Tyr and bicyclic-Phe in tumor-bearing mice. (**A** and **B**) Biodistribution of 2-F-Tyr (**A**) and bicyclic-Phe (**B**) was measured in tumor-bearing mice at 30 and 60 min following intravenous administration. Plasma concentrations, as well as tumor and small intestinal accumulations, were quantified in mice bearing B16-F10 back tumors, 30 and 60 min post-intravenous injection of equimolar doses of 2-F-Tyr (15.6 mg/kg) and bicyclic-Phe (15 mg/kg). (**C** and **D**) Tissue-to-plasma concentration ratios in tumor-bearing mice at 30 (**C**) and 60 (**D**) min after intravenous administration of 2-F-Tyr or bicyclic-Phe. T/P refers to the tumor-to-plasma ratio; I/P indicates the small intestine-to-plasma ratio. Data are expressed as mean ± S.D. for n = 4. N.S. (not significant) indicates no statistically significant difference, represented by *p* > 0.05. Differences were considered significant at *p* < 0.05.

In vitro studies revealed that bicyclic-Phe possesses high selectivity for LAT1, which prompted us to undertake further in vivo examinations. As a comparative measure, we employed 2-F-Tyr, a non-selective substrate known to be transported by both LAT1 and LAT2^40^. Given the high expression of LAT1 in tumors and LAT2 in small intestines, we evaluated the accumulation of 2-F-Tyr (Fig. 6A) and bicyclic-Phe (Fig. 6B) in tumors and small intestines in mice bearing B16-F10 tumors. After intravenous administration, measurements were taken at 30 and 60 min. The compound concentrations were measured by high-performance liquid chromatography (HPLC)^41^. Both bicyclic-Phe and 2-F-Tyr exhibited time-dependent decreases in plasma concentration, with bicyclic-Phe consistently showing higher levels than 2-F-Tyr (Supplementary Fig. S3). The concentration of 2-F-Tyr, when normalized to tissue weight for the tumor and small intestine and to volume for plasma, was higher in the tumor than in plasma at 60 min, while its concentration in the small intestine was similar to that in plasma (Fig. 6A). Conversely, the concentration of bicyclic-Phe in the small intestine was lower than in plasma, whereas, in the tumor, it was higher than in plasma (Fig. 6B). The influence of LAT1 selectivity on the tumor-specific accumulation of the compounds became apparent when calculating the tissue-to-plasma ratios.

As depicted in Fig. 6C,D, the tumor-to-plasma ratios (T/P) for 2-F-Tyr and bicyclic-Phe increased from 30 to 60 min, reaching around 2.0. The earlier attainment of this ratio by bicyclic-Phe might be attributed to its higher plasma concentration, as shown in Supplementary Fig. S3. Conversely, the small intestine-to-plasma ratio (I/P) was considerably higher for 2-F-Tyr than for bicyclic-Phe at both 30 and 60 min (Fig. 6C,D). This suggests that while 2-F-Tyr and bicyclic-Phe similarly accumulate in tumors, the small intestine preferentially uptakes 2-F-Tyr over the LAT1-selective compound, bicyclic-Phe. These findings underscore the significance of LAT1-selectivity in influencing the tissue distribution of these compounds.

## Discussion

In developing LAT1-targeted compounds, a primary objective is to achieve high selectivity for LAT1. This entails designing compounds with a strong affinity for LAT1 and minimal affinity for LAT2, a structurally similar transporter. We previously emphasized enhancing LAT1 selectivity for targeted compound delivery^5^. In this context, halogen groups are often introduced into compounds to modify ligand–protein interactions. These groups are crucial in drug design, particularly for enhancing protein–ligand binding affinity^42^. Halogen binding in protein–ligand complexes can significantly enhance binding affinity, and this enhancement tends to be more pronounced with larger halogen substituents^43^. Recent studies indicate that halogen groups at the *meta* position (position 3) in the benzene ring of Phe derivatives increase LAT1 affinity, with the inhibitory effect on LAT1 correlating with the size of the halogen group: fluorine (F) < chlorine (Cl) < bromine (Br) < iodine (I)^34^. Our findings corroborate this, showing that LAT1 prefers larger halogen groups at position 3 of halogenated phenylalanines (Fig. 2E). Additionally, we found that LAT1 binding affinity to these derivatives is influenced by the halogen group's size and position. Remarkably, the iodo group at position 2 exhibited high LAT1 affinity, comparable to position 3 and superior to position 4 (Fig. 2A). A homology model of LAT1 proposes that lipophilic groups at position 3 of Phe analogs enhance binding affinity by interacting with LAT1's hydrophobic subpocket^34^. Our findings suggest a similar hydrophobic subpocket in LAT1 that robustly interacts with halogen groups at position 2 of halogenated phenylalanines, particularly larger halogens, potentially leading to high binding affinity (Fig. 2A,C). In contrast, LAT2 does not display a marked preference for halogen groups based on size at positions 2, 3, and 4 (Fig. 2B), maintaining relatively constant affinity regardless of halogen size at position 2 (Fig. 2D). These insights underscore the importance of halogen size and position in enhancing LAT1 specificity and are pivotal for developing LAT1-selective compounds.

To better understand the properties of Phe analogs for LAT1 targeted delivery, we assessed their LAT1 affinity (*K*_i_), LAT1 selectivity (*K*_i_ ratio of LAT2 to LAT1), and LAT1 transport velocity (*V*_max_ of efflux). This study revealed that 2-I-Phe significantly enhances LAT1 affinity compared to the parent amino acid Phe, mainly due to its large iodo group at position 2 in the benzene ring (Fig. 3A and Table 1). Although 2-I-Phe demonstrated a similar LAT2 affinity and efflux *V*_max_ to Phe (Figs. 3B and 5D; Tables 1 and 2), it exhibited considerably higher LAT1 selectivity than Phe (Table 1). However, despite being a LAT1 substrate, 2-I-Phe showed a lower efflux *V*_max_ than Phe (Fig. 5C and Table 2). This reduced *V*_max_ might be due to its high LAT1 affinity, potentially impeding dissociation from the LAT1 binding site^23^. Furthermore, the bulky side chain of 2-I-Phe could inhibit LAT1's conformational changes necessary for efficient substrate transport^44^. This phenomenon mirrors our previous findings that bulky side chains can increase LAT1 affinity while reducing transport velocity^22,23^. Such a trend is also observed in the case of melphalan, an anticancer drug known for its high LAT1 affinity and low transport velocity, attributed to its bulky side chain^22–24,35,36^. These findings underscore the importance of considering both affinity and transport efficiency when developing LAT1-targeted compounds. Therefore, the goal in developing such compounds should be to maintain a high transport velocity to ensure effective delivery of the compound to the target site without compromising on the required high affinity for LAT1.

In this study, a comparison of the *K*_m_ values derived from efflux experiments for the Phe analogs revealed a trend similar to the *K*_i_ values obtained from inhibition experiments (Tables 1 and 2). This is because the efflux of radiolabeled pre-loaded LAT1 substrates, induced by extracellularly applied compounds, reflects transport mediated via LAT1. However, we observed an unexpectedly low *K*_m_ value for the efflux of bicyclic-Phe in LAT2. This discrepancy might be attributed to the residual presence of endogenous LAT1 in HEK293-LAT2 cells. A similar phenomenon was noted with FAMT, a compound known for its exclusive transport via LAT1 and avoidance of LAT2 in *Xenopus* oocyte expression systems^40,45^. Yet, FAMT induced a low level of l-[^14^C]leucine efflux in LAT2-stably expressing culture cells, where endogenous LAT1 was not completely replaced by LAT2^40^. In this study, we observed that the *K*_i_ value of bicyclic-Phe for LAT2 is approximately 3.8 mM, while its *K*_m_ value in the efflux experiments in HEK293-LAT2 cells is around 30 μM, similar to its *K*_m_ for LAT1 (Tables 1 and 2). This lower efflux* V*_max_ in HEK293-LAT2 cells induced by bicyclic-Phe (a quarter of that of Phe) is likely due to a component mediated by LAT1. Notably, α-methyl-Phe showed a higher l-[^14^C]alanine efflux level in HEK293-LAT2 cells compared to bicyclic-Phe (Fig. 5B,D, and Table 2). This suggests that α-methyl-Phe is transported by LAT2 with low affinity in addition to its transport via LAT1 in these cells.

α-Methyl-Phe, characterized by an added methyl group to its α-carbon (Fig. 1), demonstrates a preference for LAT1 over LAT2, aligning with findings from our previous research^26^. This study further confirms its higher selectivity for LAT1 compared to Phe (Fig. 3 and Table 1). However, it is important to note that the α-methyl modification, while enhancing selectivity, significantly reduces its affinity for LAT1 (Fig. 3 and Table 1). Our prior investigations have highlighted that effective LAT1 substrate binding entails interactions between the substrate's α-carboxy and α-amino groups with the transporter's binding site^23^. We noted a correlation between LAT1 binding affinity and the carbonyl oxygen charge in substrates. This was evidenced by a sequence of *K*_i_ values: Tyr < l-DOPA < α-methyl-Tyr < α-methyl-DOPA, which mirrored the increasing carbonyl oxygen charge (−0.5635 < −0.5633 < −0.5579 < −0.5558)^23^. This trend suggests that the α-methyl modification in aromatic amino acids raises the carbonyl oxygen charge (reducing its negative charge), thereby diminishing LAT1 affinity. Our current study further revealed that the α-methyl moiety does not significantly affect efflux's *V*_max_ through LAT1 (Fig. 5C and Table 2). Comparable *V*_max_ values for both α-methyl-Phe and its parent compound Phe suggest that the LAT1 binding site can accommodate the α-methyl group. However, the α-methyl modification, which elevates the carbonyl oxygen charge, appears to diminish LAT1 affinity (Fig. 3A and Table 1). This reduced affinity may hinder the effectiveness of α-methyl aromatic amino acids in LAT1-targeted delivery. Considering the competition with endogenous amino acids for LAT1 transport, compounds that exhibit high affinity for LAT1 are advantageous to ensure effective transport^25^. Our data also showed that LAT2 exhibits minimal interaction with the α-methyl moiety, displaying very low affinity and efflux *V*_max_ for α-methyl-Phe (Tables 1 and 2). Consequently, α-methyl-Phe emerges as a LAT1-selective compound characterized by high transport velocity but comparatively lower affinity.

Bicyclic-Phe is a cyclic amino acid analog in which the α-methylene moiety, marked with an asterisk in Fig. 1, corresponds to the α-methyl moiety of α-methyl-Phe. Our study has established that bicyclic-Phe exhibits significant selectivity for LAT1 (Fig. 3 and Table 1). While its LAT1 affinity is lower than that of Phe, the affinity of bicyclic-Phe is higher than that of α-methyl-Phe (Fig. 3A and Table 1). This suggests that the α-methylene moiety in bicyclic-Phe may have a reduced impact on lowering affinity compared to the α-methyl group. Moreover, our findings indicate that LAT1's binding site accommodates the α-methylene moiety of bicyclic-Phe, as its *V*_max_ of efflux was comparable to that of Phe and α-methyl-Phe (Fig. 5C and Table 2). In contrast, it appears that LAT2 does not interact effectively with the α-methylene moiety of bicyclic-Phe, similar to the α-methyl group, leading to markedly lower LAT2 affinity and *V*_max_ of efflux (Tables 1 and 2). The α-methylene moiety in bicyclic-Phe, functioning as an α-methyl mimic, notably influences LAT2's binding site. Based on these observations, bicyclic-Phe emerges as a promising candidate for LAT1-targeted delivery. It offers higher LAT1 affinity and selectivity than α-methyl-Phe while maintaining a transport velocity comparable to the parent compound Phe.

In our study, we included Phg, the basic structure of Phg-mustard^37^, in our investigation into LAT1-selective compounds. Phg-mustard emerged as a compound of interest in improving the transport velocity of Phe-mustard (melphalan). Although melphalan is selective for LAT1, it has a lower affinity for this transporter and is transported at a slower velocity compared to endogenous LAT1 substrates. It has been demonstrated that Phg-mustard can trigger the efflux of pre-loaded [^3^H]Phe via LAT1 similarly to endogenous substrates like leucine^37^. Seeking to enhance nitrogen mustard delivery through LAT1, Phg-mustard was found superior to melphalan in terms of transport velocity. To better understand its potential in developing more effective LAT1-targeted compounds, we included Phg in our study to evaluate its selectivity towards LAT1. Our findings indicate that Phg's selectivity for LAT1 is comparable to that of the endogenous substrate Phe. Notably, Phg's lack of a β-carbon diminishes its affinity for LAT1 and LAT2 (Fig. 3 and Table 1).

In the in vivo experiments shown in Fig. 6, we evaluated the biological significance of LAT1 selectivity in tissue-specific delivery of compounds. We compared the distribution of the LAT1-selective bicyclic-Phe with the non-selective 2-F-Tyr, which is transported by both LAT1 and LAT2^40^. Our observations revealed that the tumor-to-plasma concentration ratio was similar for both 2-F-Tyr and bicyclic-Phe, approximately 2.0 at 60 min (Fig. 6D). Notably, the small intestine-to-plasma concentration ratio for bicyclic-Phe was significantly lower than that for 2-F-Tyr (Fig. 6D). This observation underscores the significance of LAT1-selectivity for targeted compound delivery in vivo, given that cancer cells predominantly express LAT1^5,14^, while the small intestine exhibits extensive LAT2 expression^9,10,12^. Clinically used drugs such as gabapentin^21^, l-DOPA^17^, and melphalan^46^ utilize LAT1 for targeted delivery across the BBB or to cancer cells. However, they often face limitations such as low transport velocity, affinity, or selectivity^21–23,26^. Remarkably, a study using an in situ brain perfusion method revealed that bicyclic-Phe, crossing the BBB via LAT1, had a significantly higher transport velocity and affinity compared to melphalan. Specifically, bicyclic-Phe's *V*_max_ was approximately seven times higher, and its *K*_m_ was about 21 times lower than melphalan^47^. This indicates that bicyclic-Phe crosses the BBB with notably higher affinity and transport velocity, making it an excellent candidate for LAT1-targeted delivery to both tumors and the brain^47^. Given these findings, bicyclic-Phe emerges as a highly promising LAT1-targeted compound, potentially effective for both tumor and brain applications.

In conclusion, our study established that the halogen group at position 2 in the benzene ring of Phe and its larger size are crucial for LAT1 selectivity, though they can negatively impact transport velocity in LAT1. Furthermore, our findings demonstrated that both α-methylated Phe and bicyclic-Phe exhibit high LAT1 selectivity and efficient transport velocity, with bicyclic-Phe displaying a higher affinity than α-methyl-Phe. We advocate for a development strategy that optimizes LAT1 affinity, selectivity, and transport efficiency in targeted compound delivery. A standout candidate, bicyclic-Phe, emerged for its high LAT1 affinity, selectivity, and transport velocity, making it a promising option for targeted delivery in both tumors and the brain. These insights are instrumental in designing LAT1-targeted drug delivery systems that can selectively accumulate in tumors or the brain, maximizing therapeutic efficacy while minimizing side effects in non-target tissues.

## Materials and methods

### Materials

l-[^14^C]Leucine (338 mCi/mmol) and l-[^14^C]alanine (56 mCi/mmol) were acquired from Moravek Biochemicals (Brea, CA, USA) and American Radiolabeled Chemicals (St. Louis, MO, USA), respectively. α-Methyl-l-phenylalanine (α-methyl-Phe), (*R*)-2-amino-1,2,3,4-tetrahydro-2-naphthoic acid (bicyclic phenylalanine, bicyclic-Phe), and l-phenylglycine (Phg) were purchased from Sigma-Aldrich Chemical (St. Louis, MO, USA). 3-Bromo-l-phenylalanine (3-Br-Phe) was obtained from Angene International Limited (Nanjing, China). 2-Fluoro-l-tyrosine (2-F-Tyr) was from Amatek Chemical (Jiangsu, China). Additional phenylalanine analogs were purchased from Chem-Impex International (Wood Dale, Illinois, USA). The chemical structures of the compounds used in this study are shown in Fig. 1.

### Cell culture

Mouse melanoma cell line B16-F10 (CRL-6475) was obtained from American Type Culture Collection (ATCC; Manassas, VA, USA). HEK293 cells stably expressing human LAT1 and human LAT2 (designated as HEK293-hLAT1 and HEK293-hLAT2, respectively) were established as described previously through transfection with plasmids encoding human LAT1 and human LAT2^26^. B16-F10 cells were cultured in RPMI-1640 medium (Nacalai Tesque, Kyoto, Japan)^48^. The HEK293-hLAT1 and HEK293-hLAT2 cells were maintained in MEM medium (Nacalai Tesque, Kyoto, Japan) supplemented with 1% non-essential amino acids (Wako, Osaka, Japan) and G418 disulfate (0.9 g/L, Nacalai Tesque, Kyoto, Japan)^26^. Both cell media were supplemented with 10% fetal bovine serum (FBS, Nichirei Biosciences Inc., Tokyo, Japan) and a combination antibiotic consisting of 100 units/mL penicillin and 100 μg/mL streptomycin (Nacalai Tesque, Kyoto, Japan). Cells were incubated at 37 °C in a humidified atmosphere containing 5% CO_2_.

### Inhibition experiments

The inhibition experiments were performed as described previously^26^. HEK293-hLAT1 (1.5 × 10^5^ cells/well) or HEK293-hLAT2 (2 × 10^5^ cells/well) cells were plated onto poly-d-lysine-coated 24-well plates (Corning, New York, USA). Following a 2-day culture period, the uptake of 1 μM l-[^14^C]leucine or l-[^14^C]alanine was measured for 1 min in the absence or presence of non-radiolabeled test compounds at concentrations of 10, 30, and 100 μM. Radioactivity was quantified using a β-scintillation counter (LSC-3100; Aloka, Tokyo, Japan), and protein concentrations were determined with a Micro BCA Protein Assay Kit (Thermo Fisher Scientific, Rockford, IL). To calculate *K*_i_ values, the concentration-dependent uptake of l-[^14^C]leucine or l-[^14^C]alanine was analyzed in the presence and absence of test compounds^49^. The data were plotted against substrate concentration and fitted to Michaelis–Menten kinetics. *K*_i_ values were derived using nonlinear regression analysis with the enzyme kinetics inhibition module in GraphPad Prism software (Version 10.0.2; GraphPad Software Inc., San Diego, CA, USA).

### Efflux measurements

Efflux experiments were performed as described previously^50^. Cells cultured 48 h were pre-loaded with 5 μM l-[^14^C]leucine or l-[^14^C]alanine for 10 min at 37 °C. The cells were then washed, and the efflux was induced by incubating cells for 1 min in either the absence or presence of non-radiolabeled test compounds at indicated concentrations. The efflux time of 1 min fell within the linear range of time-dependent substrate efflux mediated by LAT1 and LAT2 (Supplementary Fig. S2). Following incubation, the medium was collected and radioactivity was measured in both the medium and the cells. Efflux was quantified as the percentage of radioactivity released into the medium, calculated using the formula: radioactivity of medium/(radioactivity of medium + radioactivity of cells) × 100%.

The kinetic parameters for the efflux of l-[^14^C]leucine or l-[^14^C]alanine induced by various concentrations of extracellularly-applied test compounds were analyzed^40^. The baseline efflux values—those obtained in the absence of test compounds—were subtracted from the values measured in the presence of the compounds. The resultant data were fitted to Michaelis–Menten kinetics to determine the *K*_m_ and *V*_max_ by nonlinear regression analysis using “enzyme kinetics-velocity as a function of substrate” module within GraphPad Prism software.

### Animal experiments

All animal experiments were performed in accordance with the guidelines of the Research Institutes of Animal Experimentation and approved by the Animal Experiments Committee of Osaka University. This study complied with the Animal Research: In Vivo Reporting of Experiments (ARRIVE) guidelines. Upon the blood sampling, mice were euthanized by exsanguination through a cardiac puncture under deep anesthesia. Anesthetization of mice was performed by intraperitoneal injection of a three-agent mixture anesthetics (midazolam 4.0 mg/kg, medetomidine 0.3 mg/kg, and vetorphale 5.0 mg/kg in saline).

To determine the plasma concentration–time profile in normal mice, female C57BL/6J mice (6–7 weeks, 15–20 g) were intravenously administered with equimolar doses of 2-F-Tyr (15.6 mg/kg/200 μL) or bicyclic-Phe (15 mg/kg/200 μL) via the tail vein. The blood samples were collected at 15, 30, 60, and 120 min post-administration into lithium heparin-coated tubes (BD, New Jersey, USA) and then centrifuged at 1,500 ×*g* for 10 min at 23 °C. The resulting plasma was stored at −80 °C pending analysis by HPLC.

In the B16-F10 tumor model, the differential accumulation of 2-F-Tyr and bicyclic-Phe in tumors was assessed compared to the small intestine. Female C57BL/6J mice were subcutaneously injected with 2 × 10^6^ B16-F10 cells. The cells were prepared in a mixture containing a 1:1 volume ratio of phosphate-buffered saline (PBS) and Matrigel (Corning, New York, USA), with the total injection volume being 200 μL, administered into the dorsal flank. Tumor growth was measured bi-daily using calipers until tumors reached a size of approximately 300 ~ 500 mm^3^, at which point they were deemed palpable. Mice were then intravenously administered equimolar doses of either 2-F-Tyr (15.6 mg/kg/200 μL) or bicyclic-Phe (15 mg/kg/200 μL) via the tail vein. Blood samples were collected at 30 and 60 min post-administration. Then both the tumors and small intestines were excised and weighed for subsequent analysis by HPLC.

### HPLC analysis

HPLC analyses were conducted as previously described^41^. In brief, tumors and small intestines were homogenized in ice-cold PBS (3 times the tissue weight, v/w). The homogenates were then centrifuged at 15,000 ×*g* for 40 min at 4 °C. A 10 μL aliquot of the tissue supernatant or plasma was mixed with 190 μL of acetonitrile (MeCN) to precipitate proteins. This mixture was subsequently centrifuged at 15,000 ×*g* for 30 min at 4 °C. The resulting supernatant (100 μL) was dried, and the residue was reconstituted in 20 μL of 400 mM sodium borate (pH 8.0). Finally, 5 μL of 40 mM 4-fluoro-7-nitro-2,1,3-benzoxadiazole (NBD-F) in MeCN was added for fluorescence derivatization. HPLC analysis was carried out using a NANOSPACE SI-2 HPLC system (Shiseido, Tokyo, Japan) equipped with a fluorescence detector. The separation was achieved on a Capcell Pak C18 MGII S5 column (250 × 2.0 mm i.d.). The mobile phase consisted of MeCN, water, and trifluoroacetic acid (TFA) in a 27.5:72.5:0.05 (v/v/v) ratio, delivered at a flow rate of 200 μL/min. Gradient elution was employed over 90 min for 2-F-Tyr and 100 min for bicyclic-Phe. Each sample, with an injection volume of 10 μL, was analyzed, and quantification of 2-F-Tyr or bicyclic-Phe was achieved by comparing the peak height of each analyte with that of a corresponding standard.

### Statistical analysis

All experiments were performed in 3–4 replications. Statistical differences were determined using the unpaired two-tailed Student’s *t*-test. Differences were considered significant at *p* < 0.05.

### Supplementary Information

Supplementary Information.

## Data Availability

All data generated or analyzed during this study are included in this published article (and its supplementary information files).
