# Supplementary material for: Structure–activity characteristics of phenylalanine analogs selectively transported by L-type amino acid transporter 1 (LAT1)
Source: Sci Rep. 2024 Feb 26;14:4651. doi: 10.1038/s41598-024-55252-w (PMC10897196; doi:10.1038/s41598-024-55252-w)
Supplement: Supplementary file 1 — Supplementary Information. [file 41598_2024_55252_MOESM1_ESM.pdf]

Structure-activity characteristics of phenylalanine analogs selectively transported by L-type amino acid transporter 1 (LAT1)

Sihui Chen[1], Chunhuan Jin[1], Ryuichi Ohgaki[1,2], Minhui Xu[1], Hiroki Okanishi[1], and Yoshikatsu Kanai[1,2,3*]

[1]Department of Bio-system Pharmacology, Graduate School of Medicine, Osaka University, 2-2, Yamadaoka, Suita, Osaka 565-0871, Japan

[2]Integrated Frontier Research for Medical Science Division, Institute for Open and Transdisciplinary Research Initiatives (OTRI), Osaka University, Suita, Osaka 565-0871, Japan

[3]Premium Research Institute for Human Metaverse Medicine (WPI-PRIMe), Osaka University, Suita, Osaka 565-0871, Japan

[*]Corresponding author ykanai@pharma1.med.osaka-u.ac.jp

Supplementary Figure S1

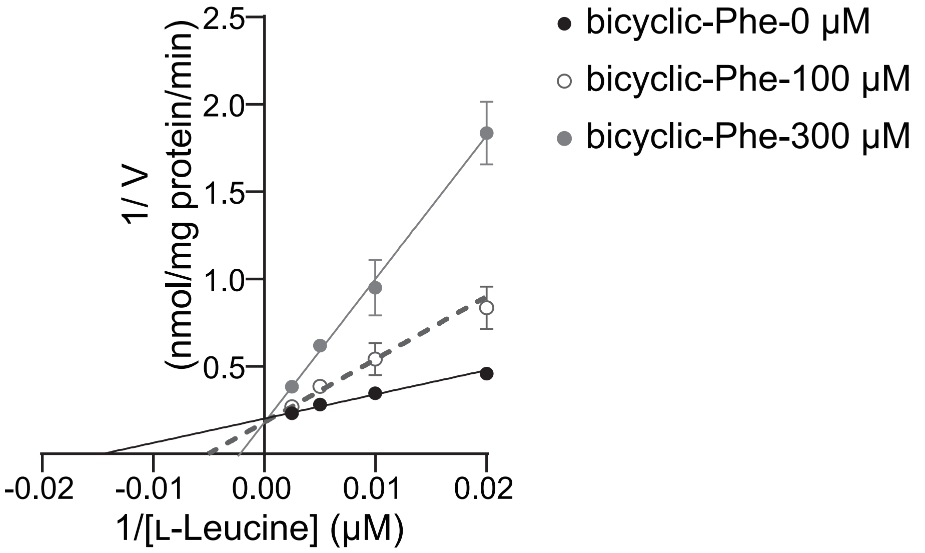

Supplementary Figure S1. Inhibition kinetics of bicyclic-Phe on LAT1. This figure presents a representative Lineweaver-Burk plot for analyzing the inhibition kinetics of bicyclic-Phe on L-[^{14}C]leucine uptake in HEK293-hLAT1 cells. The uptake of L-[^{14}C]leucine at concentrations of 50, 100, 200, and 400 μM was measured in the presence of bicyclic-Phe at concentrations of 0 μM (closed black circles), 100 μM (open gray circles), and 300 μM (closed gray circles). Data are expressed as mean ± S.D. (n = 3). The y-intercepts were not altered at different concentrations of bicyclic-Phe, confirming that bicyclic-Phe inhibits LAT1-mediated L-[^{14}C]leucine uptake in a competitive manner and the K_i values are independent of the compound concentrations; the K_i values calculated at 100 μM and 300 μM of bicyclic-Phe were 78.18 ± 13.94 μM and 66.68 ± 7.08 μM, respectively.

Supplementary Figure S2

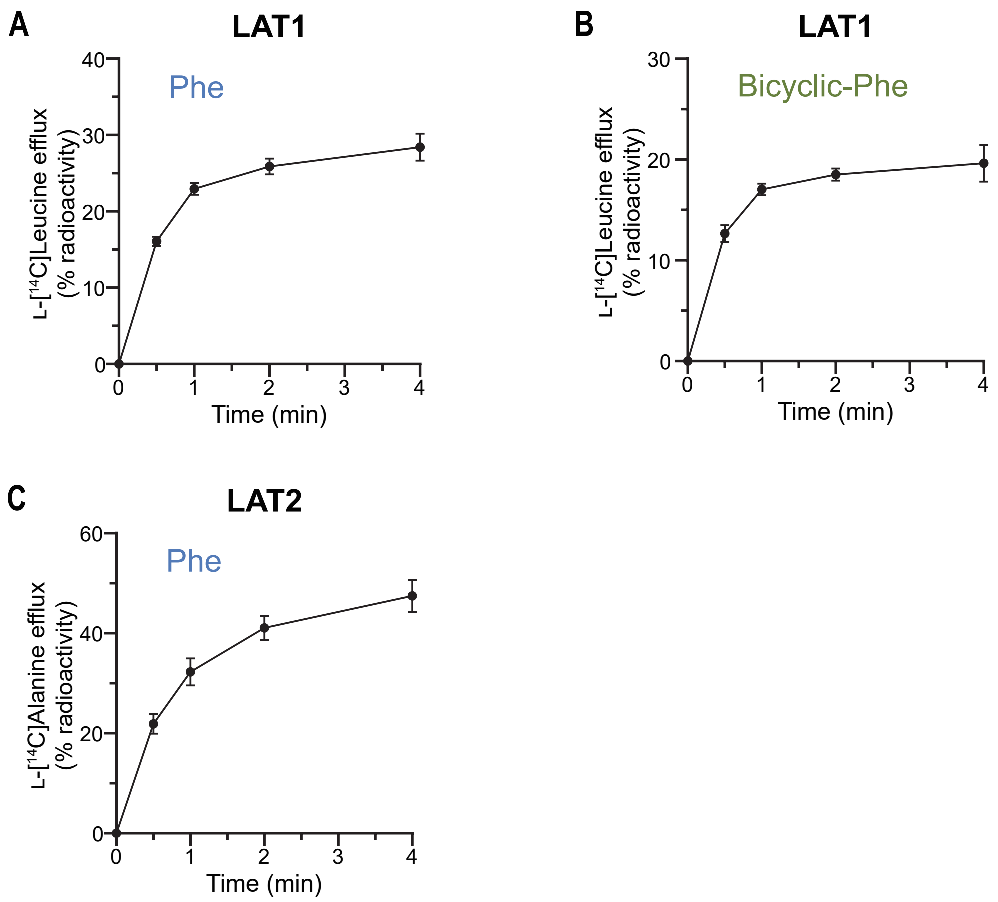

Supplementary Figure S2. Time-dependent efflux of L-[^{14}C]leucine via LAT1 and L-[^{14}C]alanine via LAT2, induced by Phe and bicyclic-Phe. Panels **A** and **B** show the time-course of efflux of L-[^{14}C]leucine from HEK293-hLAT1 cells induced by 10 μM Phe and 10 μM bicyclic-Phe, respectively. Panel **C** depicts the efflux of L-[^{14}C]alanine from HEK293-hLAT2 cells induced by 25 μM Phe. Efflux measurements were conducted over a period from 0 to 4 min in the presence of either Phe or bicyclic-Phe in the extracellular medium. Efflux values are expressed as a percentage of the pre-loaded L-[^{14}C]leucine or L-[^{14}C]alanine. Data are presented as mean ± S.D., n = 3. For kinetic analyses and comparisons between test compounds, the efflux time, based on the time course of efflux of pre-loaded substrates, was set to 1 min, which falls within the linear range of time-dependent substrate efflux.

Supplementary Figure S3

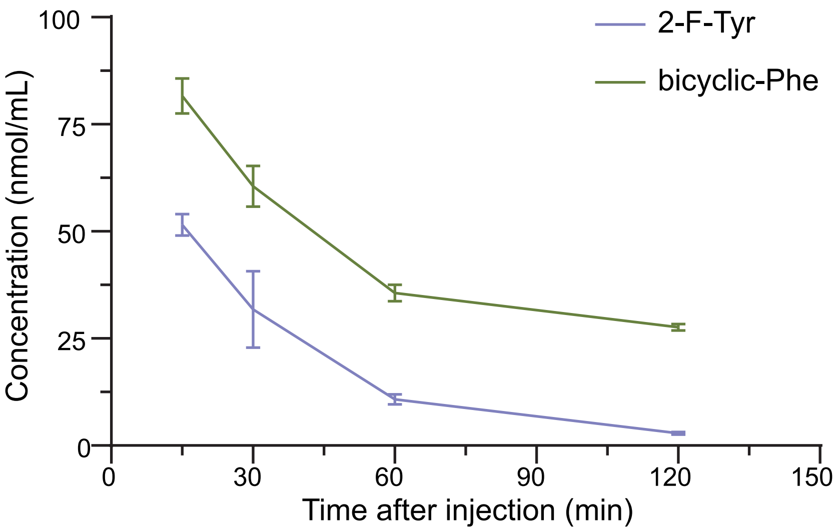

Supplementary Figure S3. Plasma concentration-time profile of 2-F-Tyr and bicyclic-Phe after intravenous administration. Plasma concentrations were measured at 15, 30, 60, and 120 min after intravenous administration of 2-F-Tyr (15.6 mg/kg) or bicyclic-Phe (15 mg/kg) into C57BL/6J mice. Data are presented as mean ± S.D., n = 3.
